# Erratum: Protein kinase C-delta (PKCδ), a marker of inflammation and tuberculosis disease progression in humans, is important for optimal macrophage killing effector functions and survival in mice

**DOI:** 10.1038/mi.2017.108

**Published:** 2017-12-20

**Authors:** S P Parihar, M Ozturk, M J Marakalala, D T Loots, R Hurdayal, D Beukes, M Van Reenen, D E Zak, S K Mbandi, F Darboe, A Penn-Nicholson, W A Hanekom, M Leitges, T J Scriba, R Guler, F Brombacher

**Keywords:** Monocytes and macrophages, Inflammation, Biomarkers, Tuberculosis

**Correction to:** *Mucosal Immunology* (2017); advance online publication, 23 August 2017; doi:10.1038/mi.2017.68

Panel f of Figure 1 was inadvertently mislabeled during production: “Cavity cecum” should read “Cavity caseum” and “Caseous cecum” should read “Caseous caseum.” The corrected figure appears below.

**Figure 1:**
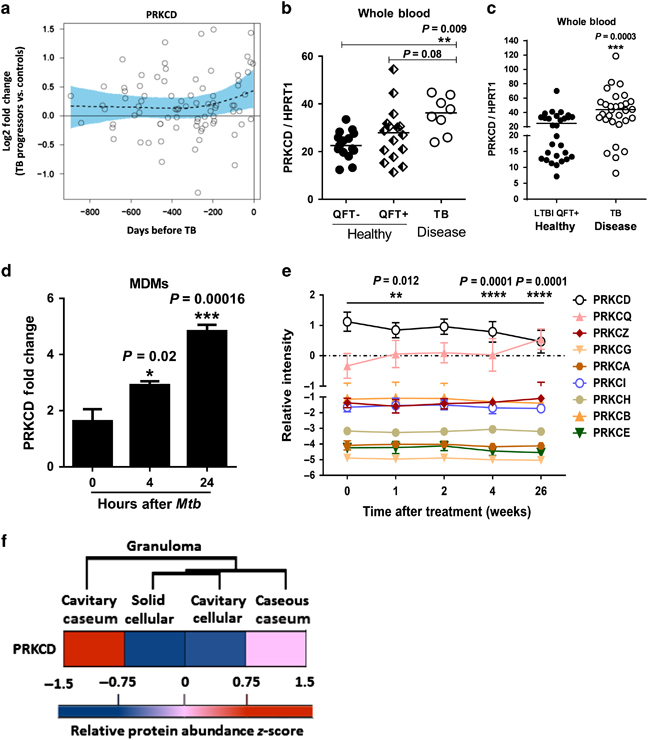
. PowerPoint slide

The publisher regrets the error.

## PowerPoint slides

PowerPoint slide for Fig. 1

