# Supplementary figures and images for: Erratum: Protein kinase C-delta (PKCδ), a marker of inflammation and tuberculosis disease progression in humans, is important for optimal macrophage killing effector functions and survival in mice
Source: Mucosal Immunol. 2017 Dec 20;11(2):579–80. doi: 10.1038/mi.2017.108 (PMC8127809; doi:10.1038/mi.2017.108)

## Slide 1
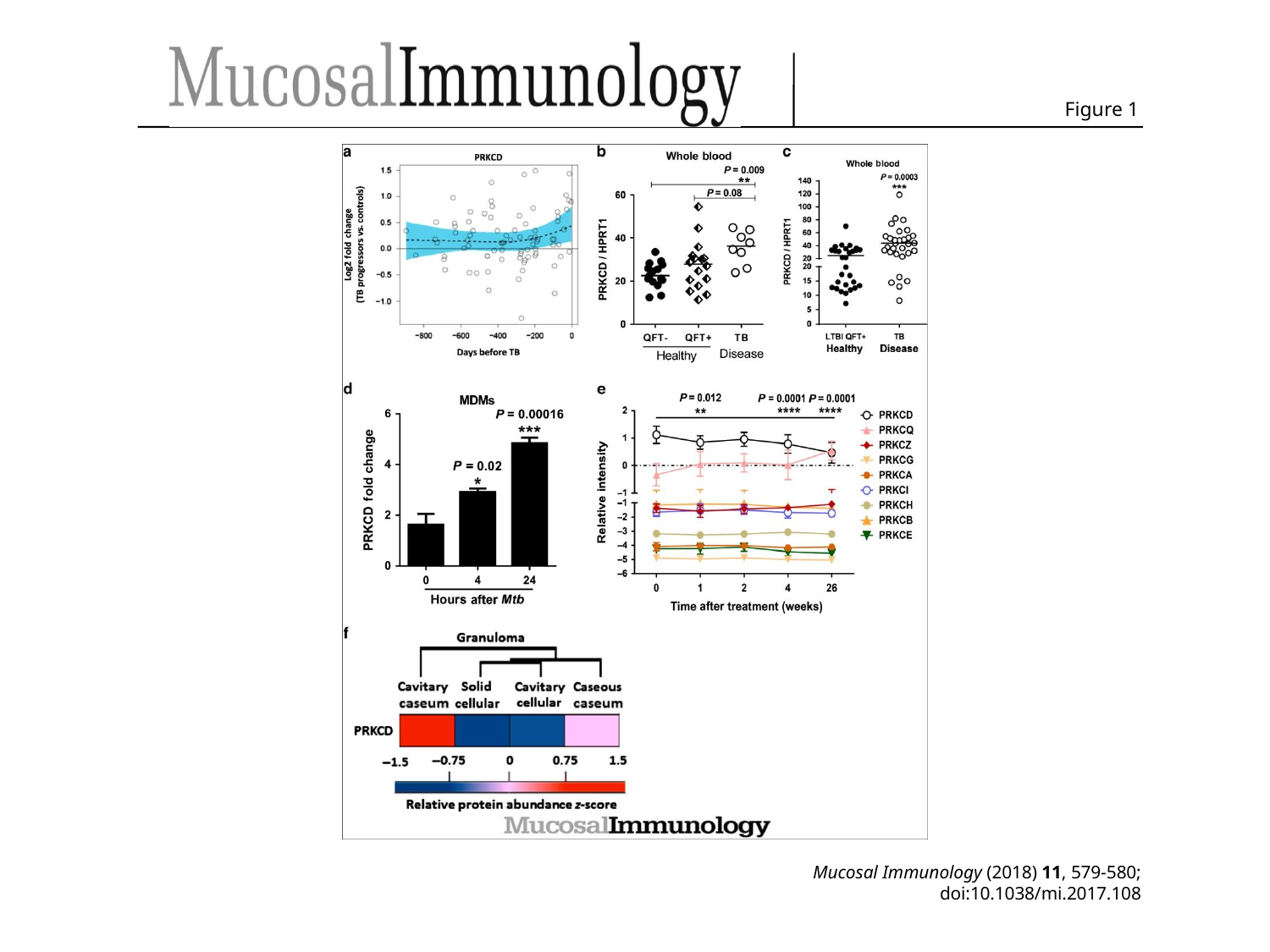

Figure 1
Mucosal Immunology (2018) 11, 579-580;
doi:10.1038/mi.2017.108

Supplement: Supplementary file 1 — PowerPoint slide for Fig. 1 [file 41385_2018_BFmi2017108_MOESM198_ESM.ppt]
